# Evaluation of cellular and molecular impact of zearalenone and *Escherichia coli* co-exposure on IPEC-1 cells using microarray technology

**DOI:** 10.1186/s12864-016-2830-z

**Published:** 2016-08-09

**Authors:** Cornelia Braicu, Sonia Selicean, Roxana Cojocneanu-Petric, Raduly Lajos, Ovidiu Balacescu, Ionelia Taranu, Daniela Eliza Marin, Monica Motiu, Ancuta Jurj, Patriciu Achimas-Cadariu, Ioana Berindan-Neagoe

**Affiliations:** 1 Research Center for Functional Genomics, Biomedicine and Translational Medicine, “Iuliu Hatieganu” University of Medicine and Pharmacy, Marinescu 23 Street, No. 23, Cluj-Napoca, 400012 Romania; 2 Department of Functional Genomics and Experimental Pathology, The Oncological Institute “Prof. Dr. Ion Chiricuta”, Republicii Street, No. 34-36, Cluj-Napoca, 401015 Romania; 3 Department of Physiopathology, Faculty of Veterinary Medicine, University of Agricultural Sciences and Veterinary Medicine, Calea Mănăștur 3-5, Cluj-Napoca, 400372 Romania; 4 Laboratory of Animal Biology, National Institute for Research and Development for Biology and Animal Nutrition, Calea Bucuresti No. 1, Balotesti, Ilfov 077015 Romania; 5 Department of Surgery, The Oncology Institute “Prof. Dr. Ion Chiricuta”, Republicii Street, No. 34-36, Cluj-Napoca, 401015 Romania; 6 Department of Surgical Oncology and Gynaecological Oncology, “Iuliu Hatieganu” University of Medicine and Pharmacy, 8 Babeş Street, Cluj-Napoca, 400012 Romania; 7 Research Center for Functional Genomics and Translational Medicine, “Iuliu Hatieganu” University of Medicine and Pharmacy, 23 Marinescu Street, Cluj-Napoca, 40015 Romania

**Keywords:** Co-exposure, *E. coli*, Intestinal epithelial cells, Microarray, Zearalenone

## Abstract

**Background:**

The gastrointestinal tract is the primary site of toxin interaction, an interface between the organism and its surroundings. In this study, we assessed the alteration of intestinal mRNA profile in the case of co-occurrence of zearalenone (ZEA), a secondary *Fusarium* metabolite, and *Escherichia coli (E. coli)*, on the intestinal porcine epithelial cells IPEC-1. We chose this model since the pig is a species which is susceptible to pathogen and mycotoxin co-exposure.

**Results:**

After treating the cells with the two contaminants, either separately or in combination, the differential gene expression between groups was assessed, using the microarray technology. Data analysis identified 1691 upregulated and 797 downregulated genes as a response to *E. coli* exposure, while for ZEA treated cells, 303 genes were upregulated and 49 downregulated. The co-contamination led to 991 upregulated and 800 downregulated genes. The altered gene expression pattern was further classified into 8 functional groups. In the case of co-exposure to ZEA and *E.coli,* a clear increase of proinflammatory mechanisms.

**Conclusions:**

These results demonstrate the complex effect of single or multiple contaminants exposure at cellular and molecular level, with significant implications that might lead to the activation of pathological mechanisms. A better understanding of the effects of co-contamination is mandatory in developing novel exposure regulations and prevention measures.

## Background

The diseases caused by mycotoxin exposure can display acute or chronic effects, the latter being the result of low-dose intake over a longer period of time, resulting in decreased productivity and reduced resistance to pathogens [1]. Chronic ingestion of mycotoxins is also a concern relating to the health of human populations [1, 2]. The gastrointestinal tract (GIT) is a major site where mycotoxins exert their effect, being the primary site of interaction [3], and as such, is frequently exposed to various toxic agents. Considering this, during the last decades the role of this GIT in primary immune defense has been intensively studied [4, 5].

The rapid uptake of ingested mycotoxins in the circulation points to the fact that these substances are mostly absorbed in the upper part of the GIT [1, 6]. Also, some mycotoxins undergo enterohepatic recirculation [1, 2], thus being present for long times in the intestine [7]. An effect of mycotoxins on the GIT can be observed even at concentrations which do not exert any systemic effects, suggesting that the combination of various mycotoxins at non-toxic individual levels may become toxic at intestinal level [6, 8].

Zearalenone (ZEA) is a secondary metabolite of certain *Fusarium* species, synthesized through a polyketide pathway by *F. graminearum, F. culmorum, F. equiseti, F. crookwellense,* etc. [9] [10]. These molds are regular contaminants of cereal crops worldwide [11]. ZEA contamination almost always co-occurs with other *Fusarium* toxins [12], such as deoxynivalenol (DON) or fumonisins B1, their combination having most likely an enhanced toxic effect compared to the individual ones [1, 13–15]. ZEA is stable and resistant to standard decontamination procedures, and can be found in processed cereal products such as beer, flour, soybean and bread [15, 16]. A particularity of this mycotoxin is that the structure of ZEA is similar to that of beta-estradiol; therefore it activates estrogen receptors [15]. This mycotoxin is frequently involved in reproductive disorders of farm animals, the most susceptible one being the pig, but is also implicated in human hyperestrogenic syndromes [7, 17]. The exposure to this toxin during pregnancy and later, during lactation, can have reversible or irreversible effects on the offspring [18, 19]. Furthermore, the toxin has been proven to have effects which are independent of its binding to the estrogen receptors [8], such as cytotoxicity, genotoxicity, immunotoxicity, and hepatotoxicity [15, 19]. For example, ZEA induces oxidative stress in a dose-dependent manner in the Caco-2 cell line [18], the SHSY-5Y cell line [20], but also in *in vivo* conditions [11, 15].

Up to 90 % of this mycotoxin is absorbed in the upper part of the GIT, and it goes into enterohepatic circulation, as is the case of other mycotoxins [1]. One of the important roles of the GIT is its function as an immune barrier. This function is accomplished through a number of particularities. Firstly, it possesses its own immune system, and it is estimated that up to 70 % of the immune defenses of the organism are located in the intestine [6, 7]. Secondly, its morphology contributes to its role as physical barrier through the tight junctions (TJs) formed mainly by occludin and claudin isoforms, and gap junctions (GJs) that permit the transfer of ions, nucleotides and other small molecules between adjacent cells, and are formed primarily by connexins. Last but not least, the intestinal microbiota plays a very important role in protecting against pathogen invasion [21]. The physical barrier properties of the intestine can be altered due to defective TJs and GJs. ZEA has been shown to reduce mRNA levels of occludin and claudin-4, and also the protein levels of connexin [22]. Therefore, the purpose of our study was to assess the impact of co-contamination of ZEA and *E. coli* at transcriptomic level, by using a relevant *in vitro* model for immunotoxicology [23], intestinal porcine epithelial cells (IPEC-1), and a custom design microarray experiment. The data were extrapolated to their human orthologues [24], and analyzed in the context of human health by using Ingenuity Pathway Analysis.

## Results

### Altered gene expression profiles as a response to ZEA exposure

Differential gene expression profiles for Control samples (untreated IPEC cells), versus single treatment cells (ZEA 25 μM, *E. coli*) and co-contaminated cell groups (ZEA + *E. coli*) were generated using one-color hybridization (Cy5) to custom porcine array slides, designed by Genotypic Technology, India.

During data analysis, for filtering upregulated genes, we considered a fold > 0.8 and for filtering downregulated genes a fold < − 0.8 in the ZEA treated samples. Expression fold values are provided in terms of log base 2. The microarray data analysis for the treated cells is summarized in the supplementary Table S1. In the case of *E. coli* treated cells, microarray data analysis identified 1691 upregulated and 797 downregulated genes, for ZEA treated cells, we observed 303 upregulated and 49 downregulate genes, while co-contamination led to 991 upregulated genes and 800 downregulated transcripts.

The altered gene expression pattern was further classified into 8 functional groups (Transcription factor, Signaling, Cell signaling, Proliferation, Cytokine, Interleukin, Inflammatory response, Growth factor). The results for the individual treatment (ZEA, *E. coli*) and combined treatment (ZEA + *E. coli*) are presented in Table 1.

**Table 1:** Functional classification of differentially expressed genes

Function Name	*E. coli*			ZEA			ZEA+ *E. coli*		
	Up regulated	Down regulated	Total Found	Up regulated	Down regulated	Total Found	Up regulated	Down regulated	Total Found
Transcription factor	146	26	172	16	4	20	125	20	145
Signaling	273	43	316	39	5	44	197	47	244
Cell signaling	25	5	30	2	1	3	17	5	22
Proliferation	170	20	190	20	0	20	122	23	145
Cytokine	74	6	80	4	0	4	40	7	47
Interleukin	82	6	88	2	0	2	39	5	44
Inflammatory response	62	5	67	4	0	4	33	4	37
Growth factor	104	14	118	15	1	16	65	12	77

### Gene interaction networks

The network analysis was conducted using Ingenuity Pathway Analysis software (Qiagen) and showed, in the case of the combined treatment, an increased activation of the genes related to a wide range of canonical pathways as displayed in Table 2.

**Table 2:** IPA revealed the top canonical pathways altered by exposure to ZEA, *E. coli*, respectively ZEA+ *E. coli*

Nr. Ctr.	NAME	*E. coli*		ZEA		ZEA + *E. coli*	
		*p*-value	Ratio	*p*-value	Ratio	*p*-value	Ratio
1.	iNOS Signaling	1.23E-06	9/44 (0.205)	-	-	-	-
2.	MIF Regulation of Innate Immunity	7.06E-06	8/41 (0.195)	-	-	-	-
3.	IL-12 Signaling and Production in Macrophages	8.15E-06	14/135 (0.104)	-	-	-	-
4.	Glucocorticoid Receptor Signaling	1.12E-05	20/261 (0.077)	-	-	-	-
5.	JAK/Stat Signaling	1.25E-05	10/72 (0.139)	-	-	-	-
6.	Renin-Angiotensin Signaling	-	-	3.47E-05	6/118 (0.051)	-	-
7.	P2Y Purigenic Receptor Signaling Pathway	-	-	5.73E-05	6.129 (0.047)		
8.	Ceramide Signaling	-	-	7.6E-05	5/84 (0.06)	-	-
9.	Melanocyte Development and Pigmentation Signaling	-	-	8.51E-05	5.86 (0.058)	-	-
10.	Unfolded protein response	-	-	1.77E-04	4/54 (0.074)	-	-
11.	IL-17A Signaling in Gastric Cells	-	-	-	-	1.83E-06	6/25 (0.24)
12.	IL-17A Signaling in Fibroblasts	-	-	-	-	1.47E-05	6/35 (0.171)
13.	Role of Tissue Factor in Cancer	-	-	-	-	5.45E-05	9/110 (0.082)
14.	PKCθ Signaling In T Lymphocytes	-	-	-	-	9.41E-05	9/118 (0.076)
15.	T Cell Receptor Signaling	-	-	-	-	1.34E-04	8/97 (0.082)

The main altered cellular and molecular pathways are displayed in Table 3.

**Table 3:** IPA to ranked molecular and cellular functions for exposure to ZEA, *E. coli*, respectively ZEA+ *E. coli*

Nr. Ctr.	NAME	*E. coli*		ZEA		ZEA + *E. coli*	
		*p*-value	# Molecules	*p*-value	# Molecules	*p*-value	# Molecules
1.	Cell Death and Survival	6.17E-14-2.86E-05	190	1.57E-05-9.21E-03	36	1.02E-14-3.07E-05	137
2.	Cellular Development	2.30E-13-2.77E-05	180	1.34E-06-8.91E-03	27	6.46E-19-3.72E-05	141
3.	Cellular Growth and Proliferation	2.30E-13- 2.77E-05	198	1.34E06-8.91E03	40	7.68E-20-3.72E-05	159
4.	Cell Cycle	2.72E-13-2.32E-05	80	-	-	1.61E-14-3.63E-05	72
5.	Gene Expression	2.11E-12-4.97E-06	119	-	-	-	-
6.	Cell –to-cell Signaling and Interacton	-	-	2.17E-05-9.21E-03	22	-	-
7.	Cellular Function and Maintenance	-	-	2.17E-05-9.21E-03	23	-	-
8.	Cellular Movement	-	-	-	-	5.75E-17-3.63E-05	104

**Fig. 1:**
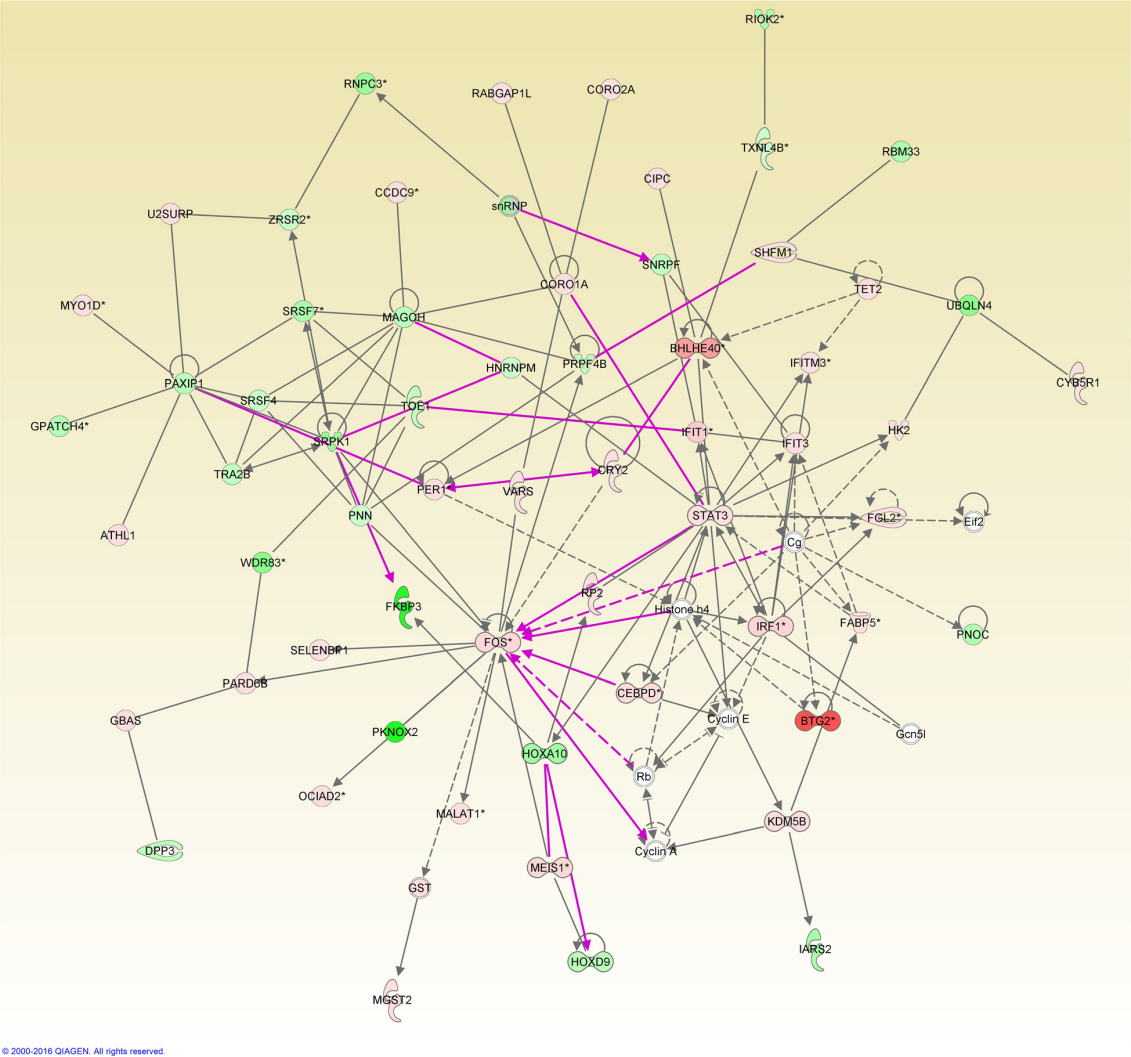
Overlay of the first two networks generated using IPA in the case of co-exposure of ZEA and *E. coli*. The red genes represent up-regulated genes and the green ones are downregulated

Figure 1 represents an overlay of the first two networks for the case of co-occurrence of ZEA and *E. coli*. presents the altered genes involved in immune-related pathways.

### Validation of the genes with an altered expression level by qRT-PCR

**Fig. 2:**
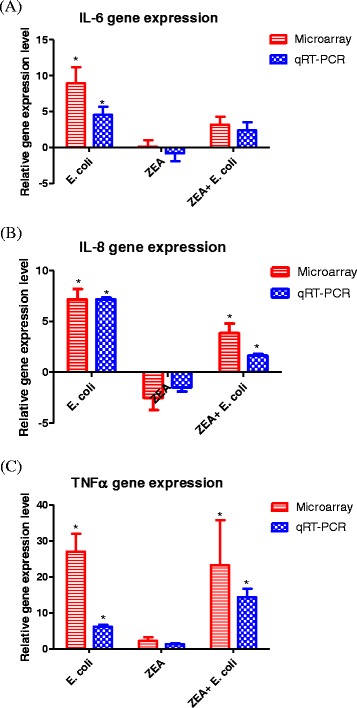
Altered gene expression level involved in the modulation of immune response related pathways for the case of **a** ZEA exposure, **b** *E. coli* exposure and **c** co-exposure of ZEA and *E. coli*. The red genes represent up-regulated genes and the green ones are downregulated (*p < 0.05, ZEA treated cells versus control cells)

The expression level of three genes, IL-6 (Interleukine 6), IL-8 (Interleukin 8) and TNFα (tumor necrosis factor alpha) was evaluated by qRT-PCR in order to validate microarray data for *E. coli*, ZEA and ZEA + *E. coli* exposure when compared to untreated cells (control) (Fig. 2). As housekeeping genes we used β-actin and CypA (Cyclophilin A). The expression levels for all evaluated three genes are in agreement with the microarray data, with slight differences in the intensity for FC.

What is important to observe is the expression level for TNFα for the combined treatment being similar with the expression level in the case of *E. coli* exposure. These suggest the activation of the fine tuning compensatory mechanisms, as a measure to contracare the co-exposure of ZEA+ *E. coli*.

## Discussions

Our study presents valuable information concerning the molecular effects of co-contamination with ZEA and another important contaminant, the bacterium *E. coli*. To our knowledge, there are only few gene profiling studies that assess the effects of co-contamination at intestinal mRNA level [1, 25, 26].

Even under normal, physiological conditions, organisms are exposed to various types of toxins and microbes, ranging from commensal microbiotas to different pathogens. But, regardless of the type of contaminant, one particularity that stands out is the fact that it is virtually impossible to find a situation where one organism is contaminated with only one agent. It is almost always a matter of co-contamination so, instead of individually looking at the effects of each toxin/pathogen, it is mandatory to look at the combined effects of all the agents that have an impact on the organism at a certain moment [6, 7, 27].

Bacterial contamination was demonstrated to cause susceptibility to carcinogenesis [28]. The complex effect of *E. coli* contamination is sustained also by our microarray data. Moreover, *E. coli* contamination was observed to cause a reduced immune response *in vitro* study, and was correlated with an increased absorption rate of mycotoxins [1, 29]. These affect the maintenance of the physiological status leading to multiple injuries, as can be observed by the large number of altered genes in the case of *E. coli* exposure.

Contamination with mycotoxins is common and virtually impossible to eradicate, especially in the case of animal fodder. Therefore, it can be stated that the effects of these mycotoxin contaminations, like in the case of ZEA, are observable also in the similar products which are consumed by humans – for instance different cereal based foods. If we also take into consideration the fact that, according to our results – as well as those of others – co-contamination is not only common, but it produces serious deleterious effects, our belief is that there is a stringent need to develop and impose strict regulations, both at national and European level, to control and counteract this phenomenon.

*E. coli* contamination has been shown to increase the absorption rates for the Fumonisin B1 mycotoxin, while other *Fusarium* metabolites were proved to increase the bacterial proliferation after oral intake of enterotoxigenic *E. coli* contaminated food by piglets [30]. The underlying mechanism seems to be an impairment of the immune function in these animals [31]. Another experiment describes an increased colonization of the porcine intestine by an Extraintestinal Pathogenic *E. coli* after exposing pigs to a one-week long diet of fumonisin B1 -contaminated food [32]. Reduced antimicrobial activity of beta-defensins against *E. coli* infection has been observed following both individual and combination treatment with ZEA and other *Fusarium sp* mycotoxins in the IPEC-1 cell line [33]. It is important see the negative effect of bacterial contamination, but these effects are more dramatic when connected with other contaminants like mycotoxins [29]. What is important is to evaluate what the threshold for the activation of the compensatory mechanism is for contracare single or multiple toxic exposures.

Following mycotoxin ingestion, the susceptibility to intestinal pathogen invasion is increased due to multiple factors [34]. The immune response is impaired as a result of mycotoxins targeting cells with a high division rate or altering the cytokine balance [34]. ZEA has been shown to influence cytokine expression both at the protein level as well as at mRNA level [4, 35], confirmed once again by the present study. ZEA had suppressive effects on the inflammatory response at the mRNA level in the BEAS-2B bronchial epithelial cell line [36], but the effect is significantly increased in the presence of *E. coli*.

**Fig. 3:**
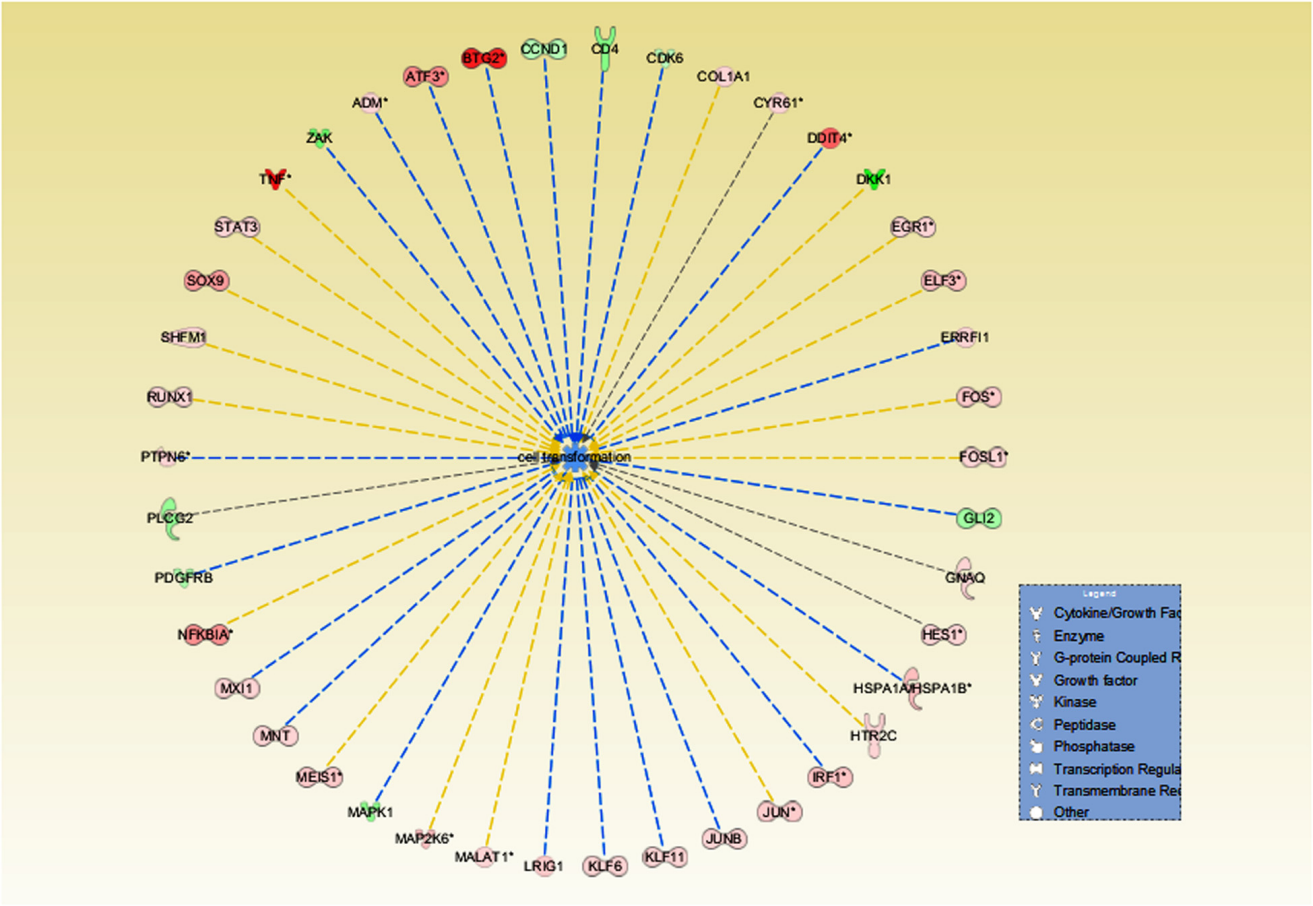
Altered gene expression level involved in the modulation of cell transformation as result of co-exposure of ZEA and *E. coli*. The red genes represent up-regulated genes and the green ones are downregulated

The microarray results that we obtained were extrapolated to human genes, in order to predict the impact of these environmental agents. Therefore, we were able to identify the top five altered canonical pathways which were related to metabolic processes in humans. A recent study on the various actions of this mycotoxin presents the immunomodulatory effect of ZEA [8]. In our research, a pathway which caught our attention was the IL-17(interleukin 17) signaling pathway. The IL-17 families of cytokines have a major role in acute and chronic inflammatory responses [37]. IL-17A is the most investigated cytokine from this family, having a pro-inflammatory role in microbial infections, autoimmune diseases, metabolic disorders and cancer [38]. The IL-17 family also has a major role in activating downstream pathways, including NFkB [39]. The activation of MAPKs and C/EBPs related pathways involve antimicrobial peptides, cytokines and chemokines, significantly activated in the case of the combined treatment. Figure 3 includes genes involved in tumorigenesis as a result of the co-exposure to the two contaminants.

In our study, the IPEC-1 cells displayed a particularly altered gene expression pattern as a result of the contaminant exposure. A significantly increased number of altered genes involved in the innate immune response, classified in “cytokines”, “interleukins” and “inflammatory response” categories, were shown to be altered (Table 1). The number of upregulated genes is roughly tenfold higher than the number of the ones that were downregulated as a result of the exposure to *E. coli*, ZEA, or the two combined. This comes in contradiction with other studies which found that this particular mycotoxin had an inhibitory effect on inflammatory responses mediated by the synthesis of cytokines and chemokines responsible for recruiting effector cells. In their study on human bronchial epithelial cells, So and colleagues observed that the mycotoxin ZEA decreased the immune response to pathogens of bacterial origin, probably through the modulation of the TLR (toll-like receptor) signaling pathway. Nonetheless, this apparent incongruity reconfirms the paradoxical rule of action of mycotoxins in general, and ZEA in particular, which states that their effect is dose- and tissue-dependent. Indeed, there are significant differences between the technical approaches of the two experiments, in what concern both the tissue type and the concentration of mycotoxin that was used [17]. Thus, while we used 25 μM of ZEA on intestinal epithelial cells, So et al., 2014 used a higher concentration, 40 μM, on human bronchial epithelial cells. The intestinal epithelial cells represent the first barrier, the interface between both nutrients and contaminants on the one hand, and the internal environment of the body on the other, being among the most important structures that contribute to the maintenance of the overall state of equilibrium in the body. On the other hand, lung epithelium represents the first outpost in the fight against air-borne pathogens [40].

Exposures to different doses of ZEA show, indeed, different types of immunological modulatory effects, in direct relation with the tissue that was analyzed. Our team presented this when conducting a series of experiments on polymorphonuclear leukocytes isolated from the blood of piglets, cultivated and treated with different doses of ZEA, ranging from 1.5 to 10 μM [35]. The *Fusarium* mycotoxin displayed clear influences on the innate immunity response of pigs, in strong relation to the particular dose that was used.

Salah-Abbes and colleagues conducted a series of experiments on blood samples, but this time from mice that were administered 40 mg kg^−1^ of ZEA, observing that the mycotoxin has an immunosuppressive effect [41]. Another example is the study of Ruh et al. on a modified, stable reporter cell line derived from murine monocytes and transfected with human estrogen receptor. Using ZEA at a concentration of 100 nM, they proved that it stimulates the promoter activity of IL1β [42].

Microarray technology has proved time and again its capacity to contribute to the characterization of genome-wide reactions to the exposure to different environmental contaminants. This was also shown by Parveen and colleagues who analyzed the estrogenic effects of ZEA on the MCF-7 human breast adenocarcinoma cell line, using the mycotoxin at a concentration of 10 nm, which proved to be optimal after several cytotoxicity and proliferation tests. Their results showed that ZEA had a similar estrogenic behavior as the natural estrogen molecule E2, even at very low concentrations, and triggered the activation of the Erk1/2 pathway [42].

## Conclusion

The co-occurrence of different contaminants represents an important issue in human health. The bacterial and mycotoxin co-occurrence is a fact commonly found in nature. The findings reported in this paper have an applicative research side, serving as knowledge base for assessing the maximum tolerance level for the ZEA mycotoxin. This is a simple example of the co-occurrence of two contaminants from the components of the exposome and the important alteration caused at cellular and molecular level. Therefore is important to evaluate these effects in the context of co-exposure, as found in real living conditions. Our study emphasizes the multiple molecular pathways altered in the case of single and multiple contaminant exposures (ZEA and *E. coli*). A significant aspect that was observed is the co-activation of the IL-17 signaling pathway and of carcinogenic mechanisms, specific for the co-contamination with *E. coli* and ZEA. This data emphasizes the complex effect of this toxin, and supports the idea to conduct further investigations in the context of co-exposure with other environmental contaminants.

## Methods

### Cell cultures and contaminants

For this study we used the primary epithelial cell line IPEC-1, obtained from the small intestine of a new-born piglet. The cells were propagated by serial passages, and incubated at 37 °C and 5 % CO_2_ in 75 cm^2^ flasks using complete DMEM/F-12 medium (Sigma) with antibiotics – Penicillin (100UI/mL) and Streptomycin (50 μg/mL), 5 % foetal bovine serum (Sigma), 2 mM L-glutamine, 15 mM Hepes (Sigma), epidermal growth factor (5 μg/L) (Sigma), insulin (10 μg/mL), transferrin (5 μg/mL) and sodium selenite (5 ng/mL) (ITS Premix, Sigma). Cells were seeded at a concentration of 2.0x10^5^ cells/well and cultivated in 24-well culture plates (Costar Coming, NY, USA). Complete confluence was obtained after 2–3 days. This cell based study is in agreement with the international tendency on replacement, refinement and reduction the animal based studies.

### Cell treatment

A solution of ZEA powder was prepared in ethanol/culture medium (1:1), then aliquoted and kept at −20 °C, and diluted in cell culture medium to assess the cellular and molecular impact.

### E. coli preparation

The K88 Enterotoxigenic *Escherichia coli* (ETEC O149) strain was used for this study. The bacteria were incubated overnight in Luria-Bertani medium (Lysogeny broth - LB) in polystyrene tubes at 37 °C and 190 rpm shaking as described by Roselli et al., (2003), then diluted in fresh broth (1:100) and incubated for 4 h. In order to determine bacterial concentration, the absorbance/optical density at 600 nm was measured (OD600). The broth was centrifuged at 4000 rpm for 10 min and the *E. coli* was harvested and resuspended in PBS. Following the adjustment of concentration, bacteria were used in the IPEC-1 cell assay.

### Bacterial and mycotoxin co-contamination

After reaching 70-80 % confluence, the IPEC-1 monolayers were treated with *E. coli* (7.0x106 CFU/ml), or 25 μM of ZEA, or a combination of the two. Distribution of the treatments was as follows: (1) control, (2) ZEA, (3) *E. coli*, (4) ZEA + *E. coli*. The treated cells were incubated for 24 h, bacteria were removed by washing, and then the cells recuperated in 0.8 ml of TRIzol Reagent (TermoFisher Scientific, Catalog number: 15596–026), then stored at -80̊C until further processing for RNA extraction, quality control and, finally, microarray evaluation.

### Microarray slide preparation

The microarray experiment was conducted using the SurePrint G3 Custom Gene Expression Microarray 8x60K (G4102A, *S. scrofa*) custom slides. The microarray probes (cRNA-Cy5) were synthesized from equal quantities of 500 ng total RNA, using the Agilent Low Input Quick Amp Labeling Kit (5190–2305) based on the manufacturer’s recommended protocol, followed by a purification step with RNeasy Mini Kit (Qiagen). cRNA-Cy5 quality control was conducted using NanoDrop ND-1000 spectrophotometer, showing a minimal yield of 1.6 μg and a specific activity of 6 pmol/μl Cy5/μg cRNA. The fragmentation and hybridization steps followed the classical Agilent protocol. After a washing step, the slides were scanned on Agilent Microarray Scanner G2565BA for low and high resolution.

### Statistical analyses of microarray data

The samples were grouped based on the replicates and the data was analyzed. The pre-processing, normalization and differential analysis of data were done using the GeneSpring GX version 12.6.1 software by the Genotypic Technology team, and the data were uploaded on the ARRAYEXPRESS database (ID: E-MTAB-3885). Lowes normalization was used to adjust the differences in intensities of the Cy5 by applying a smoothing adjustment that removes such variation. The analyses identified significant genes that were up- and down-regulated in the test samples (ZEA 25 μM) compared to the controls. Statistical *p*-value was assessed by applying Student’s *t*-test correlated with the false discovery rate (FDR – Benjamini Hochberg) correction for evaluating the impact of single and multiple compounds exposure. We considered a threshold greater and lower that 0.8 for Geomean fold for the relative gene expression level, and a *p*-value smaller than 0.05. Using GeneSpring GX Software, genes were classified according to their functionality. In order to infer the effects of ZEA and *E. coli* on human health, all the transcript sequences of *Sus scrofa* were extrapolated to their human counterparts using Homology Based Annotation retrieved from NCBI Database (www.ncbi.nlm.nih.gov), and BLAST. Ingenuity Pathway Analysis (IPA; htp://www.ingenuity.com) was applied to evaluate the impact on the biological networks that were altered as a response to different treatment scenarios.

### qRT-PCR data validation

In order to validate the microarray data, we arbitrarily selected three genes (IL-6, IL-8 and TNFα) for which we performed qRT-PCR. For the cDNA synthesis, we used an amount of 1000 ng total RNA using M-MLV Reverse Transcriptase kit (Invitrogen, Life Technologies). qRT-PCR was conducted following the method previously described by Taranu et al., 2014 [4].
